# Supplementary material for: Knowledge Network Embedding of Transcriptomic Data from Spaceflown Mice Uncovers Signs and Symptoms Associated with Terrestrial Diseases
Source: Life (Basel). 2021 Jan 12;11(1):42. doi: 10.3390/life11010042 (PMC7828077; doi:10.3390/life11010042)
Supplement: Supplementary file 1 [file life-1051735-figure S1.pdf › life-1051735-supplementary/life-1051735-figure S1.pdf]

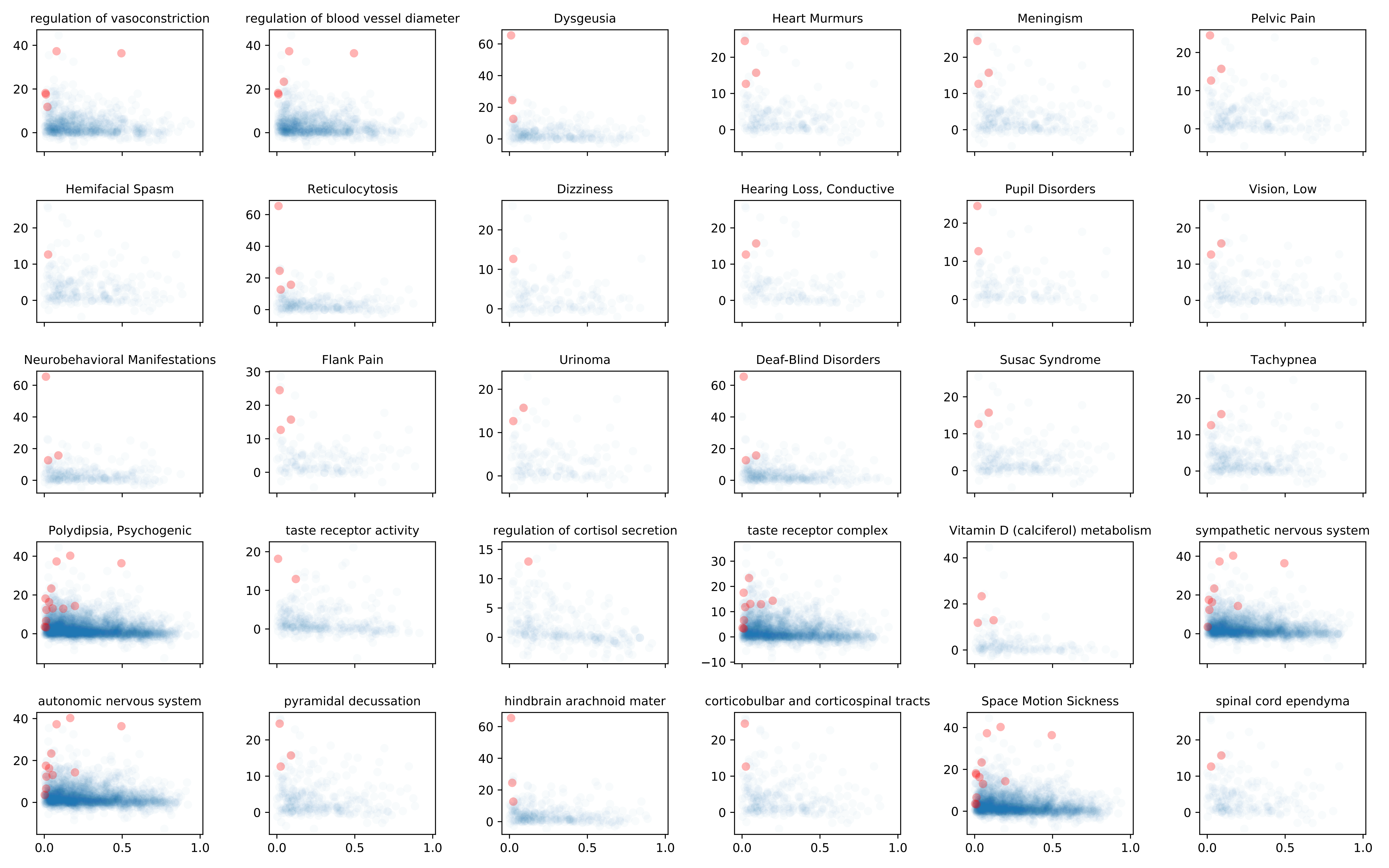

regulation of vasoconstriction
regulation of blood vessel diameter
Dysgeusia
Heart Murmurs
Meningism
Pelvic Pain
Hemifacial Spasm
Reticulocytosis
Dizziness
Hearing Loss, Conductive
Pupil Disorders
Vision, Low
Neurobehavioral Manifestations
Flank Pain
Urinoma
Deaf-Blind Disorders
Susac Syndrome
Tachypnea
Polydipsia, Psychogenic
taste receptor activity
regulation of cortisol secretion
taste receptor complex
Vitamin D (calciferol) metabolism
sympathetic nervous system
autonomic nervous system
pyramidal decussation
hindbrain arachnoid mater
corticobulbar and corticospinal tracts
Space Motion Sickness
spinal cord ependyma
